# Resource Pulses in Desert River Habitats: Productivity-Biodiversity Hotspots, or Mirages?

**DOI:** 10.1371/journal.pone.0072690

**Published:** 2013-10-04

**Authors:** Carissa L. Free, Greg S. Baxter, Christopher R. Dickman, Luke K. P. Leung

**Affiliations:** 1 School of Agriculture and Food Sciences, University of Queensland, Gatton, Queensland, Australia; 2 School of Geography, Planning and Environmental Management, University of Queensland, St Lucia, Queensland, Australia; 3 Desert Ecology Research Group, School of Biological Sciences, University of Sydney, Sydney, New South Wales, Australia; University of Florida, United States of America

## Abstract

Resource pulses in the world's hot deserts are driven largely by rainfall and are highly variable in both time and space. However, run-on areas and drainage lines in arid regions receive more water more often than adjacent habitats, and frequently sustain relatively high levels of primary productivity. These landscape features therefore may support higher biotic diversity than other habitats, and potentially act as refuges for desert vertebrates and other biota during droughts. We used the ephemeral Field River in the Simpson Desert, central Australia, as a case study to quantify how resources and habitat characteristics vary spatially and temporally along the riparian corridor. Levels of moisture and nutrients were greater in the clay-dominated soils of the riverine corridor than in the surrounding sand dunes, as were cover values of trees, annual grasses, other annual plants and litter; these resources and habitat features were also greater near the main catchment area than in the distal reaches where the river channel runs out into extensive dune fields. These observations confirm that the riverine corridor is more productive than the surrounding desert, and support the idea that it may act as a refuge or as a channel for the ingress of peri-desert species. However, the work also demonstrates that species diversity of invertebrates and plants is not higher within the river corridor; rather, it is driven by rainfall and the accompanying increase in annual plants following a rain event. Further research is required to identify the biota that depend upon these resource pulses.

## Introduction

Most hot deserts are characterized by low and uncertain rainfall, very low levels of soil nutrients and generally short and unreliable pulses of primary and secondary production [1], [2], [3]. However, many of these deserts are punctuated by drainage lines, rivers and floodplains that are thought to be more consistently productive and which receive more water than the surrounding desert areas [4]. The pulses of water in these landscape features provide habitat for aquatic and semi-aquatic organisms such as molluscs, crustaceans and fish [5], [6], [7], and also recharge ground waters that in turn allow the establishment of woody, perennial vegetation [8], [9], [10].

While much research has focused on aquatic organisms in desert rivers (e.g. [11], [12], [13], [14], more-limited ecological research has been conducted into the habitats and abiotic resources that are associated with the river channels [2], [15]. Nonetheless, several studies have described the structure of desert river habitats and suggest that they may be functionally important both as corridors and as refuges for desert biota [16], [17], [18], [19], [20]. Recent reviews underline the importance of dry riverbed habitats but also emphasize knowledge gaps about their ecological function [21], [22]. In their review of the ecology of arid Australian environments, Stafford Smith and Morton [4] suggested that the resources, habitat structure and characteristics of desert rivers differ in several important ways from those of other desert landscapes. They proposed that:

1. Floodplains and drainage lines receive more water (due to run-on) and therefore have higher soil moisture than surrounding landscapes;
2. Rivers and run-on areas have higher levels of soil nutrients (transported from run-off areas) than surrounding landscapes;
3. Perennial plants should dominate in riverine strips due to the more reliable availability of water;
4. Riverine strips should have greater plant species richness due to the greater availability of microhabitat niches than the surrounding areas;
5. Abundances and diversity of leaf-eating insects (Orthoptera, larval Lepidoptera and Hemiptera) should be greatest along river channels due to the presence of trees and shrubs; and
6. Termites (Isoptera) should be less abundant in the riverine channels than the surrounding dunes due to their preference for low-nutrient vegetation.

If Stafford Smith and Morton's [4] predictions are correct, then riverine corridors in arid regions may constitute not only a habitat for flora and fauna that are less desert-adapted, but also resource-rich refugia for ‘true’ desert biota during times of prolonged drought or climate change. Several authors have suggested that inland rivers and even palaeo-drainage channels may provide refuges for vertebrates such as mulgara (*Dasycercus cristicauda*, *D. blythi*) [23] and mala (*Lagorchestes hirsutus*) [24], and may harbour repositories of biotic diversity [20], [22]. If flows in desert rivers are driven primarily by rainfall in distant catchment areas rather than by on-site flooding, we might further expect that habitat characteristics will vary along the lengths of riparian corridors and that riparian habitats should experience stronger or more sustained pulses of productivity from ephemeral flows than are seen in the surrounding xeric landscape. There is some evidence for these expectations [18], [20], but quantitative information on the structure and functioning of desert rivers remains limited; some studies even suggest that these features are ‘mirages’ in that some biota show no response to them [25]. In a recent review, Morton et al. [26] noted that few of the predictions of Stafford Smith and Morton [4] had been tested, although there have since been considerable advances in our understanding of how water redistributes nutrients between run-on and run-off areas (e.g. [27], [28], [29]).

In this paper we describe temporal and spatial heterogeneity in the habitats and resources of a desert river system in central Australia. We selected this region because it is characterized by extreme inter-annual rainfall (and associated concentrated run-on into desert streams) [30] and hence could be expected to exhibit marked resource pulsing. Specifically, we tested the predictions of Stafford Smith and Morton [4], above, and compared soil moisture, nutrients, plant species richness, vegetation cover and invertebrates between riparian and xeric habitats and between the upper and lower reaches of the water course. We present results obtained over two and a half years that encompassed both drought and flooding rains; the rains resulted in river flows that we would have expected to temporarily accentuate any productivity differences between our study habitats.

## Materials and Methods

### Ethics statement

This study was conducted on leasehold land with permission of the leaseholder, Bush Heritage Australia. It was conducted under University of Queensland Animal Ethics Approval number SAS/610/06/UQ/US and Queensland EPA permit WISP04088306.

### Study sites and climate

**Figure 1:**
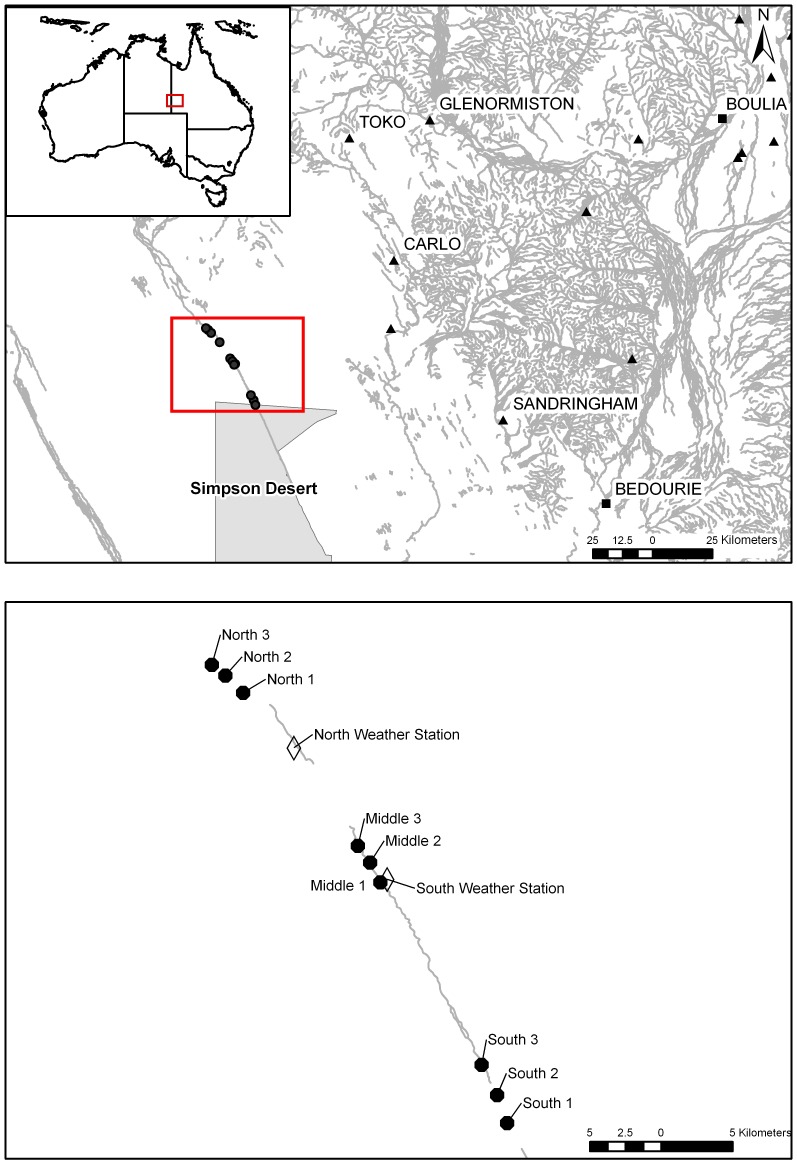
Location of homesteads (▴), settlements (▪) and the study sites (North 1–3, Middle 1–3 and South 1–3), transect lines (•) and weather stations (◊) along the Field River, Simpson Desert, Queensland.

The study was conducted along the Field River (S23°45′ E138°28′) on Ethabuka Reserve (S23°46′ E138°28′) in the north-eastern part of the Simpson Desert, Queensland (Fig. 1). The Simpson Desert is a hot sandy desert that lies within the 150 mm rainfall isopleth [31]. Ethabuka Reserve is located in the Simpson-Strzelecki Bioregion [32] and is dominated by long parallel sand dunes that run in a NNW–SSE direction [31]. The region was used for cattle grazing until 2004, but was de-stocked in that year and is now leased by Australian Bush Heritage as a conservation reserve.

The Field River originates in the Toko Ranges on the Queensland/Northern Territory border and runs for about 120 km between the sand dunes before braiding and petering out in the sand sea of the Simpson Desert [33]. Flows are ephemeral and sometimes driven by heavy on-site rainfall, but most usually by rainfall in the Toko catchment. Along the river the dominant vegetation includes *Corymbia terminalis*, *Eucalyptus camaldulensis*, *E. coolabah*, *Eremophila* spp. and *Eulalia aurea*. Vegetation in the surrounding dune fields is dominated by the hummock grass *Triodia basedowii* (hard spinifex), interspersed with perennial shrubs such as *Acacia* spp., *Grevillea* spp., *Eremophila* spp., *Sida* spp., *Dicrastylis* spp. and *Crotalaria* spp. [34], [35]; annual grasses and herbs are abundant after rain. We chose the Field River as a case study because it is representative of similar desert rivers such as the Hay, Plenty and Hale rivers further west in central Australia [36], and also because the Field has been the site of previous ecological research on vegetation [37], vertebrates and invertebrates [38], [39], [25], [40], [41].

The Field River catchment received an average of 152 mm of rainfall annually from 1995 to 2007 (recorded from two weather stations [Environdata, Warwick, Queensland] located between sampling sites established along the river channel for the present study; Fig. 1). During the study period (2006 to 2008), monthly rainfall was generally below average, but significant (337 mm) rainfall was recorded in January 2007 at both weather stations. The monthly maximum summer temperatures along the Field River ranged from 39°C to 48°C during the course of the study, with minimum winter temperatures from −5°C to 1.3°C degrees.

### Sampling design

Abiotic resources and habitat variables were measured in fixed sampling plots at three sites along the Field River. The first site (Field River South) was established on the southern border of Ethabuka Reserve (S23°58′19.3″ E138°08′31.2″), the second (Field River Middle) was 20 km to the north-northwest and the final site (Field River North) another 20 km further north-northwest still, just south of the main catchment area (Fig. 1).

In October 2005, we established three parallel transect lines of sampling plots at each site. The lines were spaced 2 km apart, and ran in a roughly east-west direction so that they intersected the river at a 90° angle. Plots were placed along each transect line, beginning beside the bank of the river and then at intervals of 15 m until there were 15 plots on each side of the river. Each plot was circular, with a radius of 2.5 m. This design allowed us to stratify the sampling of vegetation across the riverine corridor (see below) and distance from the main catchment in the northern ranges.

### Resource and habitat characteristics

**Figure 2:**
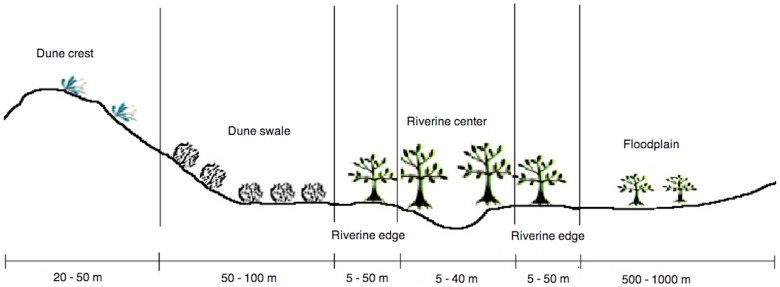
Cross sectional profile of the Field River, Simpson Desert, Queensland, showing the range and width of the five habitat types-dune crest, dune swale, riverine edge, riverine centre and floodplain. Note that this is a schematic diagram only and is not necessarily representative of the whole river.

Along each transect, sampling plots were classified as being in one of five habitat types (Fig. 2):

1. Dune crest: This comprised the top of a dune and the first 2–4 m of slope. The dominant vegetation usually consisted of shrubs and grasses *Acacia ligulata*, *A. dictyophleba*, *Grevillea stenobotrya*, *Zygochloa paradoxa*, *Dicrastylis costelloi* and *Sida* spp.
2. Dune swale: The swale was defined as running from immediately below the dune crest down slope to where the dune base levelled out and became flat. This habitat was dominated almost completely by *Triodia basedowii*.
3. Riverine edge: This was the ecotone between the central riverine woodland and the dune swale. The vegetation in this transitional habitat was a mix of *Triodia basedowii*, *Eucalyptus* spp., *Grevillea* spp. and *Acacia* spp.
4. Riverine centre: This was located on the immediate banks of the river and was dominated by tall trees including eucalypts (*Eucalyptus* spp.) and *Corymbia terminalis*. Native grasses (*Aristida contorta*, *Eulalia aurea*) were also present at most times.
5. Floodplain: Open clay floodplains occurred only in the northern study site. They were characterized by having little or no understorey and intermittent cover of mallee shrubs such as *Eucalyptus gamophylla* and *E. pachyphylla*.

To assess soil type and nutrient status, we collected soil from each of the four (middle and southern sites) or five (northern site) habitat types along each transect line using a soil corer (50 mm×50 mm square×100 mm deep). Charley and Cowling [42] demonstrated that nutrients in the arid zone are usually held in the top 5–10 cm of soil, justifying our sampling to this depth. Collection sites within habitat/lines were chosen at random, with samples coming from either side of the river channel. Three replicate samples were collected in each habitat type at each site. Soil type was assessed for clay content according to its length of ribbon and texture when manipulated, and classified by this measure (% clay) following McDonald [43]: sand (<5%), loamy sand (5%); clayey sand (5–10%); sandy loam (10–20%); loam (∼25%); silty loam (∼25%); sandy clay loam (20–30%); clay loam (30–35%); clay loam sandy (30–35%); light clay (35–40%); light medium clay (40–45%), medium clay (45–55%); and heavy clay (>55%). The percentages of soil samples in each clay category were determined for each habitat type. We used the same samples to assess soil nutrient availability, returning them to the laboratory and assaying for nitrogen and carbon content using an ‘Elementar’ vario Macro CHN/CHNS analyzer. Total available phosphorus was assayed using Kjeldahl acid digestion with sulphuric acid and copper as a catalyst [44]. The final acid extract was read using Murphy and Riley's [44] colorimetric method. Samples were collected in May 2008.

Further samples of soil were taken for soil moisture analysis in each plot using the same soil corer (50 mm×50 mm square×100 mm deep) in March, June and October 2006. Samples were placed in sealed containers and returned to the laboratory within 10 days of collection. Sub-samples were then taken, weighed and dried for 24 h to constant weight at 100°C. After drying, samples were weighed again and the percentage soil moisture calculated by the change in weight.

Assessments of ground cover and vegetation cover were made in each sampling plot on transect lines at all three sites. The percentages of ground covered by spinifex, other grasses, shrubs and forbs, trees, annual herbs and leaf litter were estimated by eye. We identified each plant species in the plots, and classified them as perennials or annuals using Urban [45] and Moore [46]. Some species could not be identified due to the condition of the plant or lack of identifying features (i.e. seeds, flowers, fruits). Unidentified plants were included in counts of species richness but not in species lists. All habitat variables were assessed at the three sites in March, June and October 2006; May and September 2007; and February and May 2008. No variables were measured at the northern site in March 2006 due to flooding.

### Invertebrate abundance and composition

Invertebrate abundance and diversity were assessed using 125 ml, clear plastic, pitfall traps. The traps were filled with 5% formalin solution and set singly in each sampling plot with the opening flush to the ground surface. Each trap was left open for three days and nights before being retrieved, with sampling carried out on the same seven occasions as the scoring of habitat variables. Captured invertebrates were classified to Order and counted in the laboratory. We chose pitfall trapping over alternative methods of invertebrate sampling, such as beat-sweeping of vegetation and digging trenches for termites [47], [48], because pilot trials in the study area showed these latter methods to yield few invertebrates for a large expenditure of effort (CRD, personal observations; [47]). By contrast, preliminary inspection of the contents of our pitfall traps indicated that they effectively captured many invertebrates, including both termites and leaf-eating insects.

### Statistical analysis

To test the predictions of Stafford Smith and Morton [4], we were interested in evaluating differences in habitat, resource characteristics and invertebrates between habitats and between sites and, for some variables, in how patterns changed over time. In the first instance, we used the general linear model (GLM) function in SAS version 9.1 [49] to explore the effect of habitat, site and the interactions of these factors on the availability of soil carbon, nitrogen and phosphorus. Samples were pooled across lines and habitats or sites for analyses, with habitat and site treated as fixed factors. Where effects were significant, we compared least-square means using *F*-tests. For other variables we computed 3-factor analyses of variance in R [50] to explore the effects of habitat, site and time. Prior to analyses of variables estimated as percentages (soil moisture, cover values of spinifex, other plant groups and leaf litter) data were subjected to angular transformation (sin ^−1^ √p); numbers of invertebrates were log-transformed (except after ants were removed and then raw numbers were used), but plant species richness was analyzed using the raw data. Because of the complexity of the analyses and many highly significant results, AIC (Akaike's Information Criterion) was employed to identify the best fitting models. This procedure provides an efficient means of measuring one model against another based on the number of parameters, standard error (residual sums of squares) and sample size [51]. AIC was defined as: AIC = N (Ln(Residual SS/N)) + (2*p) where N = sample size and p = number of parameters [52].

Invertebrate taxon composition between habitats and sites was compared using a Bray-Curtis similarity index, calculated in PRIMER-E [53], using non-transformed count data for each habitat type and site. Dendrograms were created using cluster ordination for each habitat type and site with data pooled across sampling times. The Simper procedure in PRIMER-E was used to calculate the contribution of each order to similarity between habitats.

## Results

### Resource and habitat characteristics

Soils along the riverine edge, riverine centre and in the floodplain had higher proportions of clay than those on the dunes. Samples taken from the dune crest were either loamy sand or clayey sand with no more than 10% clay content. On the other hand, most samples taken from the riverine centre contained more than 20% clay while all samples from the floodplain were classified as clay loam sandy (30–35% clay) or medium to heavy clay (>45% clay).

**Figure 3:**
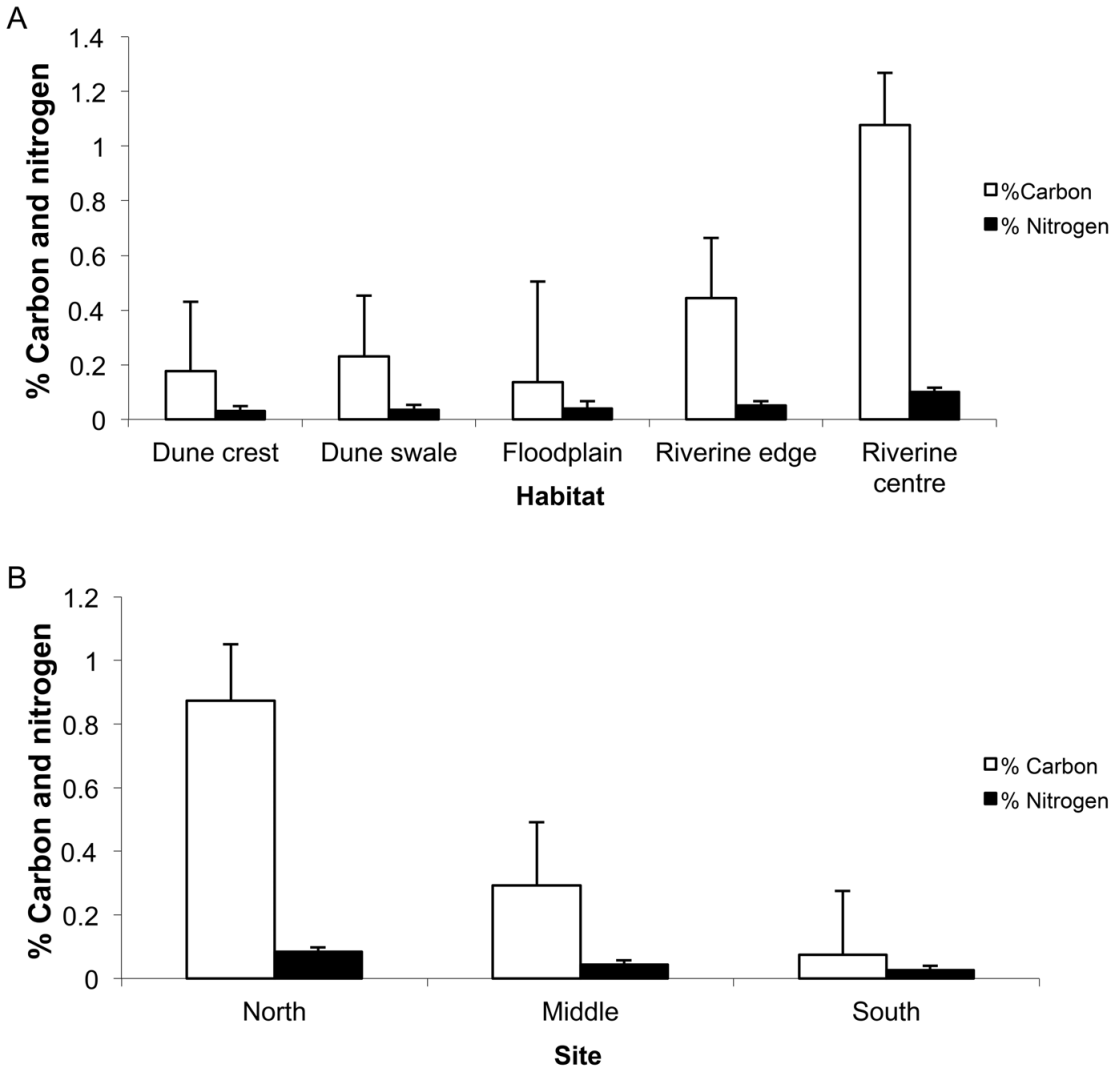
Percentage carbon and nitrogen (mean ± s.e.) in soil samples collected along the Field River, Simpson Desert, Queensland, in May 2008. Samples collected from (A) northern, middle and southern sites, (B) different habitats.

Both carbon and nitrogen levels in the collected soil samples were extremely low (on average 0.053% for nitrogen and 0.441% for carbon). Soils were, nonetheless, richer in carbon in the northern than in the middle and southern sites (*F*_2,38_ = 4.30, *P*<0.001, Fig. 3A), and in the riverine centre compared to the other habitats (*F*_4,37_ = 3.37, *P*<0.001, Fig. 3B). Soil nitrogen content showed similar trends, with higher levels in the riverine centre than in the other habitats (*F*_4,37_ = 4.22, *P*<0.001, Fig. 3B), and higher levels also in the northern than the middle (*F_4,38_ = *5.03 *P* = 0.012, Fig. 3A) and southern sites (*P* = 0.002), but no difference between the latter two sites (*P* = 0.290). The additive model of habitat + site yielded the best fit for both soil nutrients (Table 1). Only five of the 39 soil samples yielded phosphorus levels above the amount detectable by our method (0.02%). These samples had low levels of phosphorus (0.02–0.04%) and were found only in the riverine centre (north, middle and south sites) and floodplain habitat (north site only).

**Table 1:** AIC values for the effects of site, habitat and time on habitat variables measured on transects lines at three sites and in five habitats along the Field River, Simpson Desert, Queensland.

	Site (d.f = 2)	Habitat (d.f = 4)	Time (d.f = 6)	Habitat + Time (d.f = 10)	Habitat + Site (d.f = 6)	Time + Site (d.f = 8)	Habitat*Site (d.f = 13)	Habitat *Time (d.f = 34)	Time *Site (d.f = 20)	Habitat* Time + Site (d.f = 35)	Habitat *Site + Time (d.f = 18)	Site*Time + Habitat (d.f = 23)	Habitat *Site*Time (d.f = 85)	Habitat + Site + Time (d.f = 12)
Soil Moisture	−3051.6	−3077.8	−2921.0	−3132.8	−3175.2	−3113.2	−3235.1	−3126.80	−3115.0	−3245.4	**−3320.2**	−3255.267	−3305.21	−3251.5
% Carbon	−236.6	−240.3			**−243.5**		−240.9							
%Nitrogen	−21.9	−24.3			**−27.61**		−26.5							
Spinifex cover	661.1	352.4	1247.2	352.0	91.6	665.9	**−218.0**	388.08	681.12	128.33	−218.05	105.37	−118.25	93.79
Grass cover	−311.4	−672.1	−289.0	−681.3	−723.5	−317.4	−823.5	−670.02	−309.2	−722.90	**−835.91**	−729.66	−766.26	−734.3
Tree cover	−2573.0	−2624.7	−2560.4	−2635.8	−2624.8	−2584.4	−2635.7	−2606.41	−2567.0	−2607.9	**−2648.4**	−2619.90	−2564.18	−2637.1
Shrub cover	−272.4	−80.0	−8.9	−102.3	−355.7	−293.1	−103.9	−409.91	−318.3	−388.86	**−434.35**	−407.14	−409.93	−378.3
Annual cover	424.4	314.2	279.3	31.8	316.72	164.61	276.0	−138.99	59.65	−138.25	−17.21	−85.14	**−247.49**	33.76
Leaf litter	1161.8	1027.5	1435.9	918.5	794.7	1069.5	781.4	901.30	1036.6	646.45	659.63	**626.23**	661.05	674.52
Number of insects	511.5	488.5	487.1	448.5	492.0	475.3	502.1	**445.66**	468.25	449.15	461.03	**445.17**	474.78	452.11
Richness of insects	−5976.0	−5967.4	−6280.4	−6277.2	−5971.0	−6282.3	−5972.7	−6310.32	**−6359.1**	−6310.7	−6283.0	−6355.38	−6321.04	−6278.3
Richness of plants	−4656.0	−4726.0	−4799.6	−5006.4	−4758.7	−4924.	−4805.5	−5058.75	−4952.5	−5104.35	−5108.40	−5082.40	**−5187.31**	−5049.8

The best fitted model is highlighted in bold. % Carbon and % Nitrogen were not measured over time.

**Figure 4:**
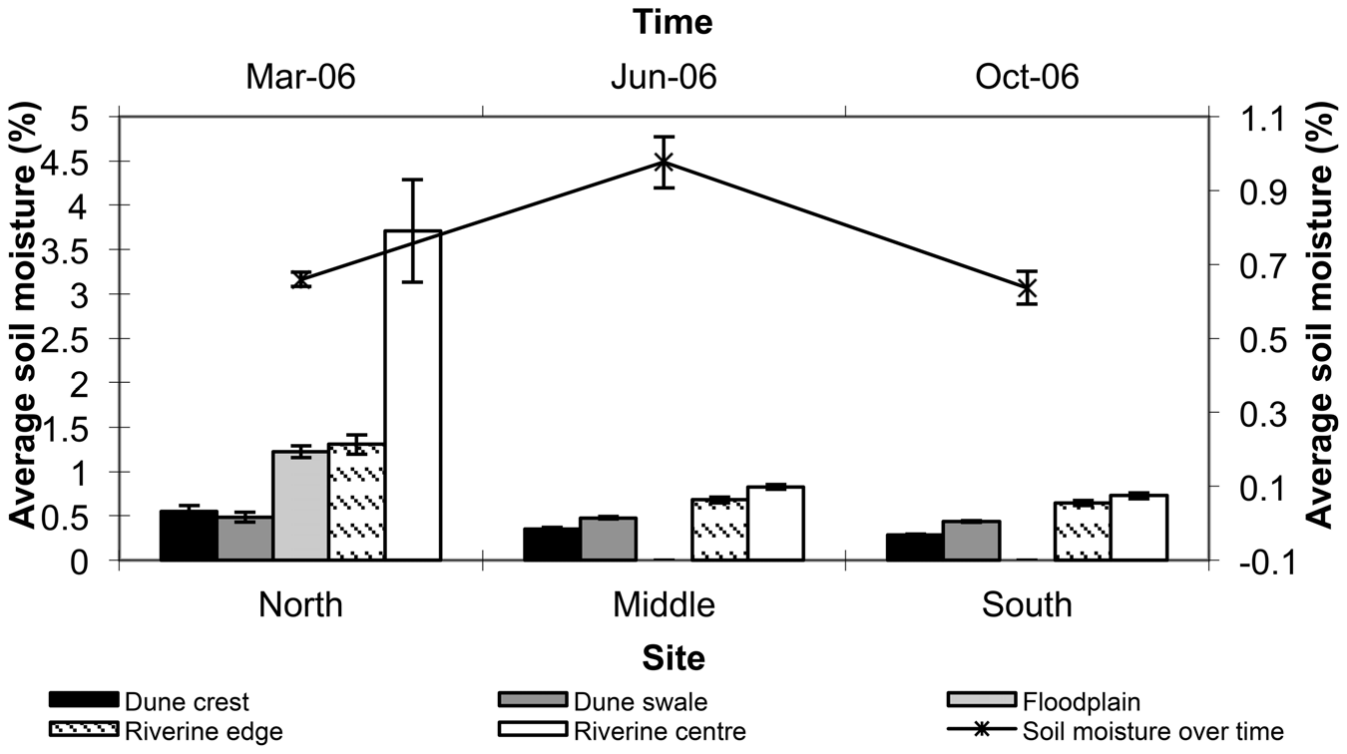
Mean (± s.e.) soil moisture (%) in five habitats at the northern, middle and southern study sitesalong the Field River, Simpson Desert, Queensland (left axis) and over time (right axis) from March 2006 to October 2006.

Soil moisture was consistently highest in the riverine centre, although the differences between habitats became much less distinct as distance from the main channel increased (Fig. 4). Not surprisingly, moisture levels were also higher at the northern than at the two more southerly sites, probably reflecting proximity to the main catchment area. The dune crest and dune swale had the lowest soil moisture at all sites. Further, soil moisture was greatest in winter (June 2006) and lowest in spring (October 2006) and summer (March 2006), giving rise to a best-fit model that incorporated all variables (Table 1).

**Figure 5:**
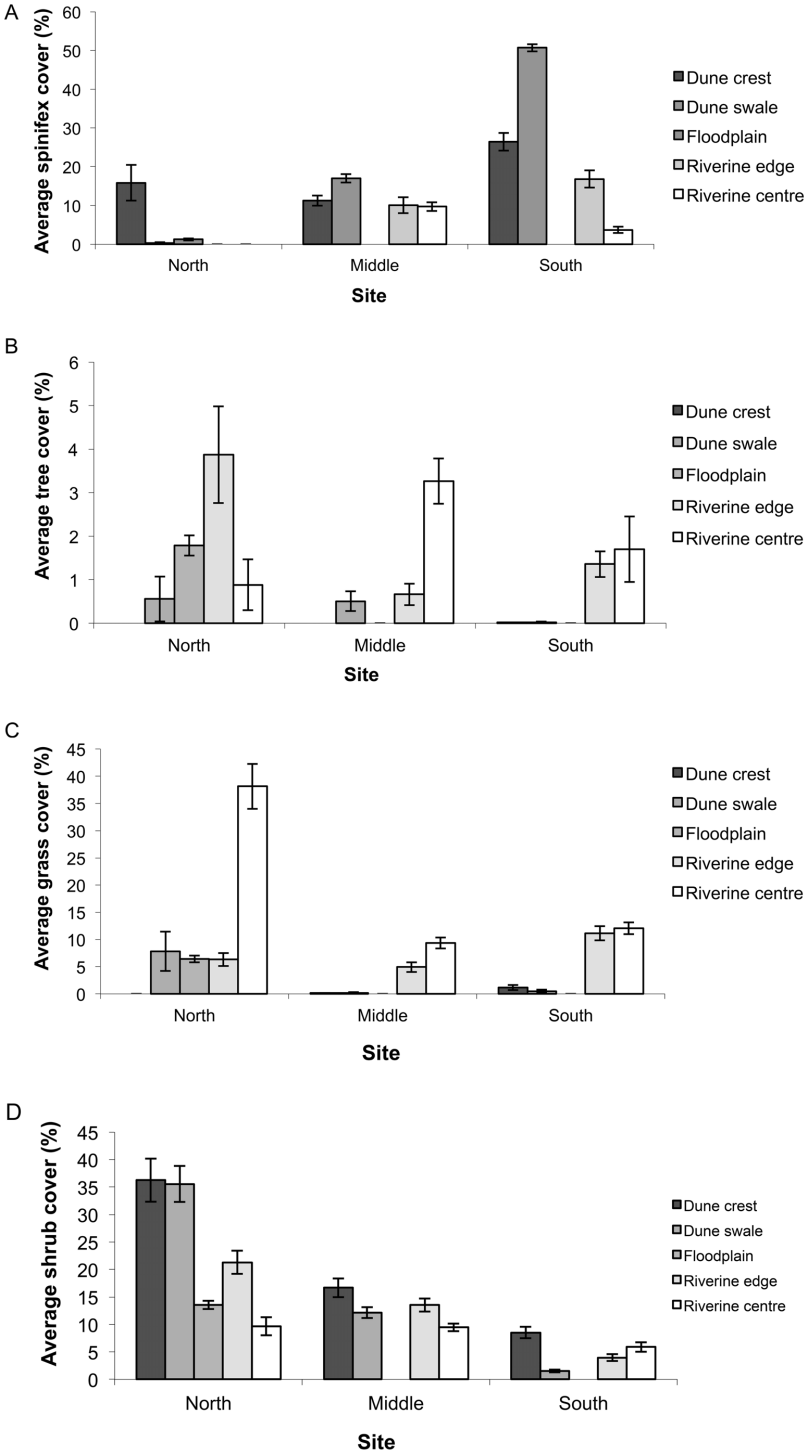
Percentage cover (mean ± s.e.) of (A) spinifex, (B) trees, (C) annual grass, and (D) shrubs at northern, middle and southern study sites in five habitats along the Field River, Simpson Desert, Queensland. In (C), average grass cover over time is shown by the line (right axis).

**Figure 6:**
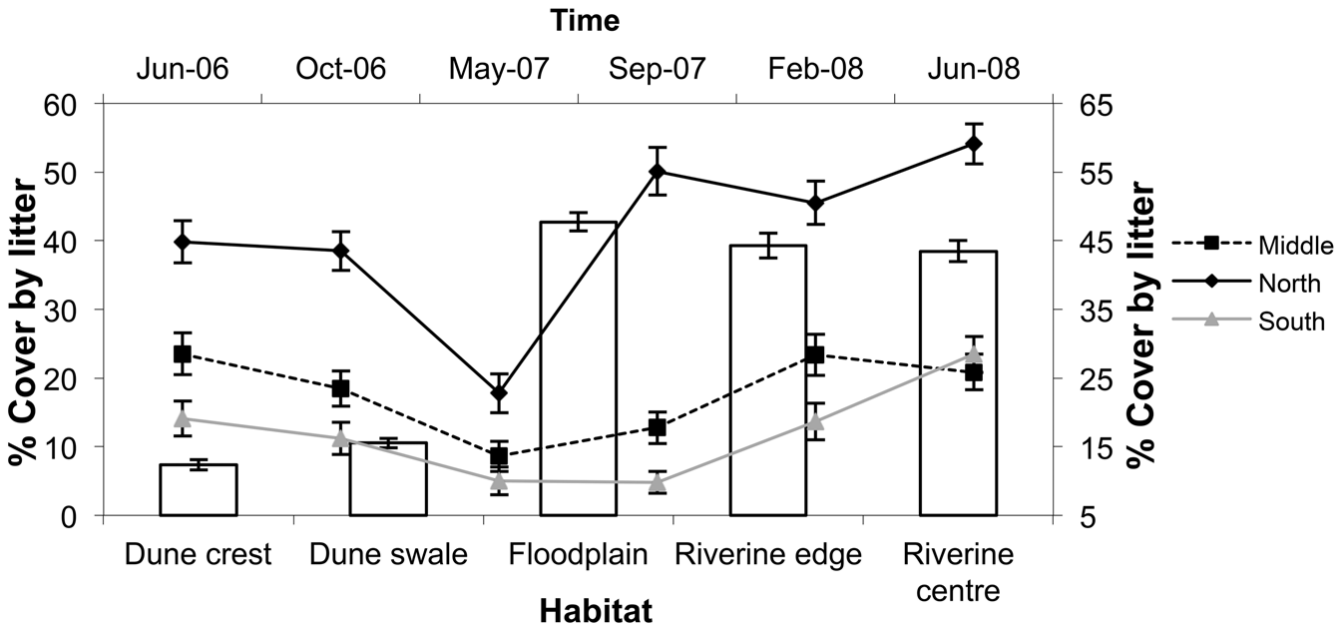
Percentage leaf litter cover (mean ± s.e.) in five habitats (bars - left axis) and in the northern, middle and southern sites (lines - right axis) from June 2006 to May 2008, along the Field River, Simpson Desert, Queensland.

Spinifex cover varied more over space than time (Table 1). In the middle and southern sites, the dune swales had more spinifex cover than the crests, riverine centre and riverine edge. The riverine centre had the lowest spinifex cover in all sites (Fig. 5A). Tree cover showed a reciprocal pattern, being greatest in the riverine centre in the southern and middle sites, but highest along the riverine edge in the northern site (Fig. 5B). Not surprisingly, tree cover was least on the dune crests and swales and generally decreased with increasing distance from the catchment. Similar site by habitat patterns of cover were displayed by annual grasses (Fig. 5C) and shrubs (Fig. 5D) with cover being generally greater in northern than southern sites, but grasses achieving higher coverage in riverine habitats and shrubs on dune crests and swales. Time was an additional factor in best fit models for the three latter variables (Table 1), with grass cover peaking in September 2007 and May 2008 (Fig. 5C) and shrub and tree cover generally increasing following the rainfall event in January 2007. The riverine habitats (floodplain, riverine centre and riverine edge) had the highest levels of leaf litter cover within sites, and the northern site had the greatest cover over all sampling sessions. Leaf litter decreased at all sites following rainfall in January 2007, but a more dramatic increase in this habitat component in the northern site in September 2007 than in the other two contributed to a habitat + site × time interaction in the best-fit model (Table 1, Fig. 6).

**Figure 7:**
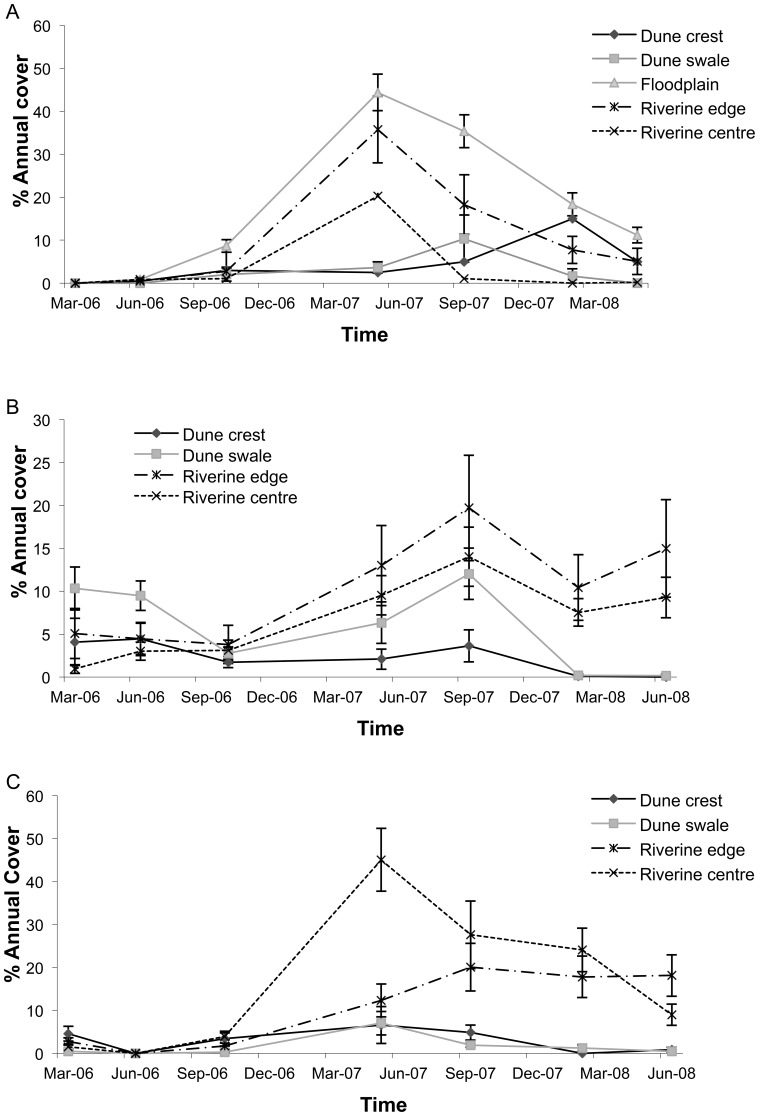
Percentage cover of annual plants (mean ± s.e.) in five habitats in A) northern, B) middle and C) southern sites, along the Field River, Simpson Desert, Queensland from March 2006 to May 2008.

There was strong spatial and temporal variation in cover of annual plants throughout the study (Table 1). Annual cover was greatest in the riverine centre and edge at the middle and southern sites, but higher on the floodplain in the north (Fig. 7A, 7B, 7C). Cover increased at all sites in all habitats after rain in January 2007. In the northern site cover of annual plants peaked at >44% in May 2007 in the floodplain and was still at >10% cover 13 months after the rain. Cover peaked in the middle site in September at 20% and in the southern site in June at 45%. Cover of annual plants in the dune habitats was generally <10% throughout the study (Fig. 7A, 7B, 7C).

Seventy species of plants were identified over the study period. More than 100 other plants were collected but it was difficult to identify many of these due to a lack of flowers or identifiable features. Fifteen species were found exclusively along the river including silky browntop (*Eulalia aurea*), *Lobelia darlingensis*, annual verbine (*Psoralea cinerea*), *Malvastrum americanum* and *Eucalyptus* spp. Perennials increased monotonically in richness from 30 species overall in dune crest habitat to 45 species in the riverine centre, with 37 found on the northern floodplain. In contrast, the richness of annual plant species was highest on dune crests (18 species), especially after the rains of early 2007, and exceeded richness in the dune swale and riverine habitats by 17–33%. Owing to the differing spatial and temporal responses of annual and perennial plants, the best fit model for plant species richness was the interaction between habitat, site and time (Table 1).

### Invertebrate abundance and composition

Approximately 725 000 invertebrates from 21 orders were collected in 5500 trap nights from 2006–2008. Most specimens belonged to the Order Hymenoptera, with ants (Family Formicidae) comprising most of these. Preliminary analysis showed that this one Order contributed 99.3% to average similarity among habitats and 97.6% between sites, so it was removed to allow exploration of compositional patterns among the less abundant remaining taxa. Invertebrates (excluding Hymenoptera) in the riverine centre, riverine edge and dune swale habitats shared ≥80% similarity, with those of the dunes crests sharing 61% similarity with these habitats and the floodplain sharing only 37% of invertebrates, on average, with all the other habitats. The Orders Collembola (springtails), Araneae (spiders) and Acarina (mites), contributed over 58% to average between-habitat similarity. Compositional similarity of invertebrates was greater between the sites, with the middle and northern sites sharing 84% of taxa and the southern site 76%, on average, with the other two.

Invertebrate numbers increased at all sites following the rain in January 2007, especially at the northern site, but fell again by May the following year. Overall, the floodplain habitat yielded more than twice the numbers of invertebrates than were found in any other habitat. Ants comprised >99% of all captures, but hemipterans, larval lepidopterans and orthopterans were caught consistently over the study. However, the abundances of these groups were extremely variable and, as standard errors often exceeded the means, there were no evident patterns in their numbers. Isopterans also were captured consistently, with a tendency for their numbers to increase both with distance from the river and distance from the northern catchment. Overall, variation in invertebrate taxon richness and numbers was explained best by models incorporating habitat with a site by time interaction (Table 1).

## Discussion

The riverine habitats differed from the dune habitats in many distinct ways, and provided strong support for the first three predictions of Stafford Smith and Morton [4]. With respect to the first prediction, soil moisture was more than twofold greater in the riverine centre and edge than on the dune crests and swales at all sites and sampling times. Soil moisture also diminished with increasing distance from the main water catchment in the Toko Ranges, presumably reflecting the progressive reduction in flow in the terminal reaches of the river. Despite these trends, soil moisture at all sites, habitats and sampling sessions was extremely low (<6%); the river did not flow during 2006 when the soil samples were taken. In the dune habitats, moisture levels did not differ greatly between sites and remained between just 0.2 and 0.4%. Soil type may explain some of the variation between riverine and dune habitats. Soil in the riverine centre, riverine edge and floodplains had more clay than the red sands of the dune crests and swales. As soils with a high clay content are known to have a high water holding capacity [54], the clay soils in the floodplain may retain moisture longer than in the sand dunes, with consequent implications for plants such as herbs and grasses.

Levels of soil carbon and nitrogen were greatest in the riverine centre habitats, thus supporting the second prediction of Stafford Smith and Morton [4] that soil nutrients accumulate in areas of run-on. Increased levels of nutrients likely result from several processes. Firstly, nutrients are transported in sediments and silts down drainage lines during floods. Jacobson et al. [55] demonstrated that soil nutrients on floodplains were highly correlated with the amount of silt in the soil. Those authors showed further that levels of soil carbon, nitrogen and phosphorus decreased downstream, as we also observed during this study. Secondly, Jacobson et al. [55] suggested that some carbon and nitrogen in floodplain soils results from the breakdown of organic matter that has built up under riparian vegetation and is buried under silt during floods. Micro-organisms and fungi break down the organic matter and release the nutrients into the soil. As leaf litter accumulated particularly in the floodplain, riverine centre and riverine edge habitats, the breakdown of this material likely contributed to the higher nutrient profiles in these habitats, which in turn contributed to the high cover abundance of annuals that we observed. Thirdly, Gutierrez and Whitford [56] found that increasing nitrogen content, in nitrogen-poor soils, increased the biomass of annuals in the Chihuahuan Desert. These authors also suggested that perennials exploit soil nitrogen by storing nitrogen in their tissues, later returning it to the soil through decomposition. Fourthly, the high clay content in the riverine and floodplain soils may act as a ‘sponge’ for holding nutrients [57], whereas sandy soils are more likely to leach nutrients into the lower-lying swales [58]. Finally, drying-wetting cycles can stimulate nitrogen mineralization and availability in ephemeral desert rivers [59], thus increasing its level in riparian soils compared to those above the riverine corridor.

Tree cover and abundance were greatest in the riverine centre, immediately above the river channel, and greater also in the northern compared to the more southerly sites. Numbers of all perennial plant species showed similar trends and, although the difference in perennial species richness between the riparian and dune habitats was not marked, these patterns still support the third prediction of Stafford Smith and Morton [4]. The woodland lining the riverine corridor consisted largely of *Corymbia terminalis*, *Eucalyptus camaldulensis* and *E. coolabah*, with trees also being much taller (>10 m) along the river than on the floodplain (<8 m) and towards the edges of the corridor (<5 m). Annual and perennial grasses, particularly *Eulalia aurea*, also dominated the riverine corridor, together with tall perennial shrubs such as *Eremophila longifolia*, *Senna artemisioides* and *Atriplex nummularia*. The more constant moisture supply and higher levels of soil nutrients probably enhance the establishment and persistence of these perennials during dry periods. In contrast to these trends, spinifex and low shrubs dominated in sand dune habitats. While the lower levels of moisture and nutrients in sandy soils may constrain shrub growth, spinifex prefers impoverished sandy soils [60] so that its dominance on dunes towards the distal reaches of the Field River was not unexpected.

Cover of annuals increased markedly after rain in January 2007, with the strongest and most sustained responses occurring on the floodplain and riverine habitats. These observations accord with our expectation that pulses of productivity should be stronger in riparian than in more xeric habitats. Indeed, on the floodplain at the northern site where the cover of annual plants was greatest, the increased cover persisted for more than 13 months after rainfall. Jacobson et al. [55] suggested that floodplain soils may hold moisture at depths greater than 30 cm for up to a year after flooding, while Gutierrez and Whitford [56] showed that supplies of water allowed annuals in the Chihuahuan Desert to emerge faster, grow for longer and increase their production of biomass. The water-holding properties of the floodplain soils in our study may similarly allow annuals to survive longer than those on the dune crests and swales, and hence increase their biomass and investment in reproduction. High biomass of annuals in turn may increase the availability of food (e.g. seeds and green vegetation) for granivorous and omnivorous animals such as rodents, many birds and invertebrates, enhancing the potential of the riverine corridor to function as a refuge or conduit for species to penetrate the otherwise xeric environment.

Despite the trends for perennial species, overall plant species richness was not consistently greater in the riverine than in the xeric dune habitats. Richness instead increased in all habitats at all sites due largely to the germination of annuals following heavy rainfall in January 2007, with the biggest increase observed on the dune crests and the most muted in the swales. These results provide little support for Stafford Smith and Morton's [4] fourth prediction that plant species richness should be greatest in riverine habitats. Several plausible explanations can be posited for this. Firstly, riparian habitats could be expected to be favoured by large grazing animals such as cattle (*Bos taurus*), camels (*Camelus dromedarius*) or red kangaroos (*Macropus rufus*), which could deplete preferred forage species. However, this seems unlikely. Cattle were present at the Field River on only 3–4 brief occasions between 1995 and 2004 (D. Smith, pers. comm.), after which they were removed entirely from the region; camels and kangaroos occur, but at densities <0.05 animals/km^2^ [61], [62], [63]. Smaller grazers such as rabbits (*Oryctolagus cuniculus*) also are present sporadically but in very low numbers [64], [65]. Secondly, and more plausibly, patches of dune crest and swale habitat, but not the riverine habitats, burnt during fires in the summer of 2001–2002 [66], and this may have facilitated seed germination and plant growth following the heavy rains in early 2007. Thirdly, and also quite plausibly, the trees and other tall perennial plants that dominate the riverine habitats may have reduced the establishment of new species through creation of shade or other forms of habitat alteration, or via competition for space and light (e.g. [67]) The perennial *Eulalia aurea* was particularly dominant in the riverine centre and edge habitats at all sites, and strong growth and flowering activity of this may have had a markedly suppressive effect on the establishment of other species. The dune habitats, particularly the crests, had fewest perennials, especially in patches that had burnt. The open sand, sparse leaf litter and exposure to extremes of temperature, rainfall and wind may have reduced competition in these habitats and allowed for rapid germination and plant succession as conditions improved.

Stafford Smith and Morton's [4] fifth and sixth predictions received little support from the results of our invertebrate sampling. With respect to prediction five, there was no evidence that herbivorous insects such as orthopterans or leafhoppers (Hemiptera: Cicadellidae) were more abundant in the riverine centre, despite the high cover there of apparently palatable vegetation. It is possible that, because we identified invertebrates only to Ordinal level, we could not distinguish whether more-specialist herbivores were present in the riverine habitats or not, or whether the habitats differed in the alpha-level diversity of insects that they support. In addition, the small numbers of potential herbivores captured (Hemiptera: 187; Orthoptera: 215) made it difficult to reliably discern any between-habitat patterns that may have existed; a further study using sampling methods aimed at orthopterans and hemipterans may be required to distinguish the habitats that they use.

With respect to prediction six, termite numbers tended to increase down river from the main catchment, but the trend was not significant. This result was surprising. Termites in general in arid environments favour low-nutrient vegetation [68], [69] such as spinifex. There was very little spinifex in the riverine habitats compared to that in the sand dunes, yet termite abundance did not reflect this pattern. It is possible that the grasses and detritus of the riverine habitats were used as alternative sources of food, irrespective of their nutrient status, thus allowing termites to extend to the river corridor. However, the sample size for termites was low (236 individuals), and this may have obscured any distributional pattern across habitats. Although pilot trials suggested that our small sampling vials would collect termites, the vials were probably more effective for surveying surface-active invertebrates than subterranean termites. Alternative methods, such as deployment of cellulose or tissue baits, again may be required to sample termites effectively [70].

Invertebrate abundance increased after rain, peaking in September 2007 at the northern and middle sites and in February 2008 at the southern site. Invertebrates were most numerous in the floodplain habitat, with ants constituting over 99% of all captures. The abundance of ants in this habitat may reflect the cover of annuals and short-lived perennials, which increased dramatically in the floodplain after the rain. Many annuals were seeding in May and September 2007 (CLF, pers. obs.), and seeds were visible on the soil surface. Many ant burrows were present on the floodplain in these months, with seeds from *Crotalaria smithiana*, *Acacia ligulata*, *A. dictyophleba* and other shrubs observed around the entrances (CLF, CRD, pers. obs.). Ants are major seed consumers and dispersers in Australian deserts [71], [72]; the pulse of seeds from annuals on the floodplain following the heavy summer rain in 2007 thus may explain the high abundance of ants that was recorded there.

## Conclusions

Ephemeral river systems occur throughout the world's arid regions and, despite being subject to limited research [15], often comprise structurally distinct habitats and act as repositories of biological diversity [22]. Our data confirm that the riverine corridor we studied is more productive than the surrounding desert, and support the idea that it has the potential to act as a potential refuge and as an access path for peri-desert species, even though we did not demonstrate an increased plant or invertebrate diversity with increased proximity to the river at the time of our sampling. Further work now is needed to describe the habitats and resources of other desert riverine systems to identify the biota that use and depend on these linear desert features, and any temporal patterns in that use.
